# *Pseudomonas aeruginosa *vesicles associate with and are internalized by human lung epithelial cells

**DOI:** 10.1186/1471-2180-9-26

**Published:** 2009-02-03

**Authors:** Susanne J Bauman, Meta J Kuehn

**Affiliations:** 1 Duke University Medical Center, Department of Biochemistry, Duke University Medical Center, Durham, NC 27710, USA

## Abstract

**Background:**

*Pseudomonas aeruginosa *is the major pathogen associated with chronic and ultimately fatal lung infections in patients with cystic fibrosis (CF). To investigate how *P. aeruginosa*-derived vesicles may contribute to lung disease, we explored their ability to associate with human lung cells.

**Results:**

Purified vesicles associated with lung cells and were internalized in a time- and dose-dependent manner. Vesicles from a CF isolate exhibited a 3- to 4-fold greater association with lung cells than vesicles from the lab strain PAO1. Vesicle internalization was temperature-dependent and was inhibited by hypertonic sucrose and cyclodextrins. Surface-bound vesicles rarely colocalized with clathrin. Internalized vesicles colocalized with the endoplasmic reticulum (ER) marker, TRAPα, as well as with ER-localized pools of cholera toxin and transferrin. CF isolates of *P. aeruginosa *abundantly secrete PaAP (PA2939), an aminopeptidase that associates with the surface of vesicles. Vesicles from a PaAP knockout strain exhibited a 40% decrease in cell association. Likewise, vesicles from PAO1 overexpressing PaAP displayed a significant increase in cell association.

**Conclusion:**

These data reveal that PaAP promotes the association of vesicles with lung cells. Taken together, these results suggest that *P. aeruginosa *vesicles can interact with and be internalized by lung epithelial cells and contribute to the inflammatory response during infection.

## Background

*Pseudomonas aeruginosa *is the major pathogen associated with chronic and ultimately fatal lung infections in patients with cystic fibrosis (CF). Current research suggests that *P. aeruginosa *live anaerobically in the mucus layer of the CF lung and are rarely found in contact with epithelial cells [1,2]. Extracellular proteases are secreted by *P. aeruginosa*, including Las A, elastase, alkaline protease, and protease IV, and these are known contributors to virulence in lung infections [3-5]. Like other gram negative bacteria, *P. aeruginosa *also release spheres of outer membrane known as outer membrane vesicles [6]. They consist of entrapped periplasmic components and outer membrane constituents, including lipopolysaccharide (LPS), glycerophospholipids, and outer membrane proteins (OMPs) [7]. Due to their small size, vesicles potentially gain access to host cells more easily than whole bacteria. Considering that vesicles are armed with bacterial proteases, toxins, surface adhesins and/or invasins, vesicles present a potentially significant contributor to lung damage caused by *P. aeruginosa*. Since they contain many immunostimulatory compounds, it is not surprising that *P. aeruginosa *vesicles induce a significant IL-8 response from cultured human lung cells [8].

Vesicles allow bacteria to disperse a complex of soluble and insoluble bacterial products into the surrounding milieu. Vesiculation appears to be a conserved process among both pathogenic and non-pathogenic bacteria and the role of outer membrane vesicles in pathogenesis is a burgeoning area of research [9]. Many pathogenic bacterial species aside from *P. aeruginosa *produce vesicles that contain toxins or other virulence factors and, in several cases, vesicles have been proposed to be vehicles for toxin delivery to eukaryotic cells [10-16]. In order to deliver toxic content, vesicles must first bind to host cells. Vesicles from *Shigella flexneri *[17], *Borellia burgdorferi *[18], *Actinobacillus actinomycetemcomitans *[13,19] and ETEC [14,20] have been observed to bind cultured host cells. Vesicles have also been observed interacting with host cells *in vivo*. In biopsies of infected patients, vesicles from *H. pylori *were found to bind intestinal cells [10,21].

*P. aeruginosa *vesicles have been amongst the most thoroughly studied vesicles to date. They have been shown to contain the virulence factors pro-elastase, hemolysin, phospholipase C, and alkaline phosphatase, as well as the penicillin-degrading enzyme β-lactamase and the *Pseudomonas *quorum sensing signal (PQS) and other hydroxyalkylquinolones [22-24]. We recently reported that the secreted aminopeptidase, PaAP, is enriched in outer membrane vesicles that we purified from each of the tested CF strains of *P. aeruginosa *[8]. Interestingly, PaAP expression was found to increase 21-fold when PAO1 was grown under microaerobic conditions [25], and 103-fold when it was grown in the presence of primary lung epithelial cells [26]. An analogous zinc metalloprotease was discovered to be associated with vesicles produced by a different CF pathogen, *Burkholderia cepacia*, and a strain with a knockout in this gene was less virulent in an animal model [27]. Such studies have stimulated much interest in determining how vesicles and vesicle components contribute to *P. aeruginosa *infection and disease in the lungs.

In this study, we use both cultured and primary airway epithelial cells to investigate vesicle-host cell interactions and to assess the contribution of PaAP to this interaction. We report that *P. aeruginosa *vesicles are internalized by human lung cells and PaAP increases vesicle association with lung cells. The results point to physiological roles for *P. aeruginosa *PaAP and vesicles during an infection.

## Results

### *P. aeruginosa *vesicle association with lung epithelial cells is strain-dependent

**Figure 1:**
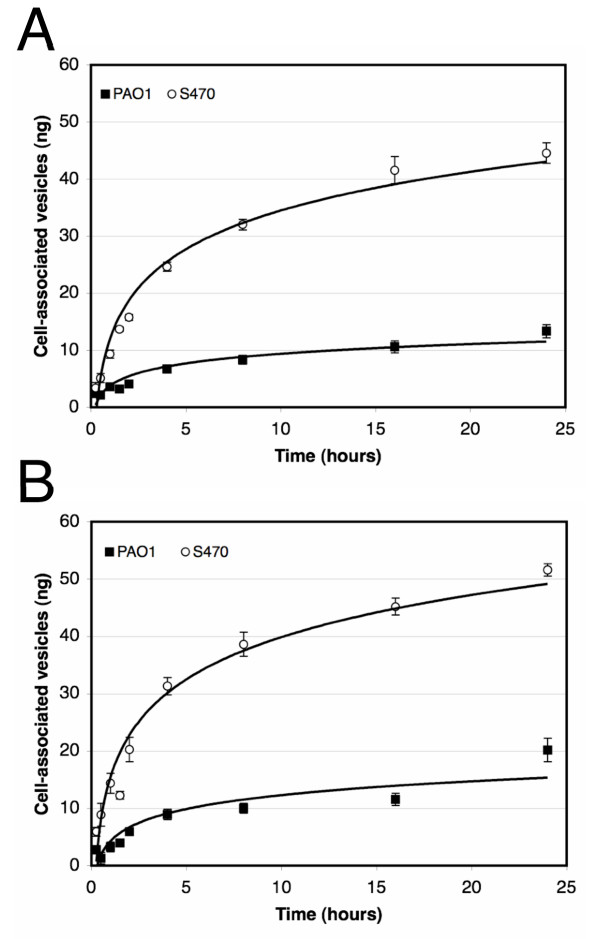
**Vesicles from a CF isolate associates to a greater extent with lung cells compared to PA01 vesicles**. FITC-labeled vesicles (2.5 μg per well) were incubated with confluent monolayers of approximately 5 × 10^4 ^A549 cells (*A*) or HBE cells (*B*) for indicated times at 37°C. Fluorescence associated with washed, solubilized cells was quantitated and correlated to vesicle amount using standard curves generated for vesicles from each strain. Experiments were done in triplicate, SEM is indicated for 2 to 7 separate experiments. At the 24 h time point, p < 0.001 for each data set.

We examined whether vesicles from various *P. aeruginosa *isolates would associate with cultured human respiratory epithelial cells. Fluorescently labeled vesicles (FITC-vesicles) from late log-phase cultures were incubated with A549 human lung epithelial cells and the amount of vesicles associated with host cells after incubation at 37°C was quantitated using a previously established microtiter plate assay [14]. To account for minor variability in the fluorescent labeling of vesicles, the amount of cell-associated vesicles was extrapolated from standard curves relating fluorescence to ng of FITC-vesicles for each of the vesicle preparations. Cell-associated fluorescence increased over time for vesicles for each of the *P. aeruginosa *isolates, however significantly more (3.3-fold) vesicles from the CF isolate S470 associated with A549 cells compared with PAO1 vesicles (Fig. 1A). The cell association profile for vesicles from another CF isolate, CF2, was very similar to the one exhibited by S470, and host cell association of vesicles from all isolates was dose-dependent (data not shown).

To test whether the vesicles would interact similarly with primary cells, we incubated vesicles with human bronchial epithelial (HBE) cells from healthy human volunteers (Fig. 1B). The results for the HBE cells were similar to those with cultured cells, thus cultured cells appeared to be a good model for primary cells in further assays. Together, these data indicate that *P. aeruginosa *vesicles from CF strains associate to a greater extent with epithelial cells than vesicles from a non-CF strain.

**Figure 2:**
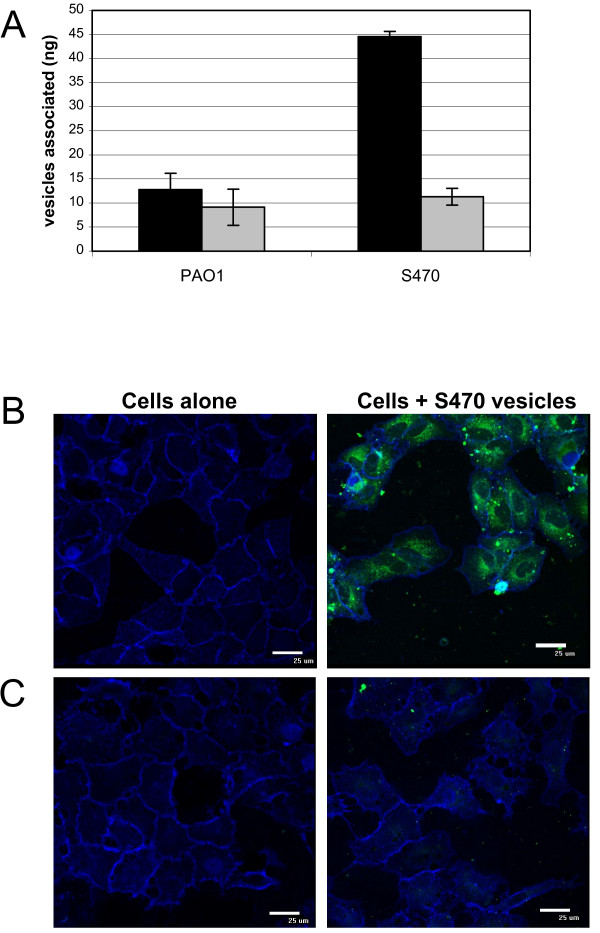
**S470 vesicle association with host cells is temperature-dependent**. A, FITC-labeled-vesicles (2.5 μg per well) were incubated with A549 cells (5 × 10^4 ^cells per well) for 24 h at 37°C (black bars), or 4°C (gray bars). SEM is indicated, n≥2, in triplicate. B and C, A549 cells alone (left panels) or incubated with 2.5 μg FITC-labeled S470 vesicles (green, right panels) for 6 h at 37°C (B) or 4°C (C). After incubation, cells were washed, labeled with AF633-WGA (blue), fixed in 2% paraformaldehyde, and visualized by confocal microscopy.

When we tested temperature dependence of vesicle-host cell association we found that incubation at 4°C substantially decreased the amount of S470 vesicles associated with the lung cells, whereas little-to-no difference was observed for PAO1 vesicles (Fig. 2A). These data indicate that a temperature-dependent mechanism was responsible for the differences observed in the association between vesicles from a CF strain and vesicles from a non-CF strain.

### *Pseudomonas aeruginosa *vesicles are trafficked into lung epithelial cells

Temperature-dependent association of S470 vesicles suggested that these vesicles may be internalized by the lung epithelial cells. We used confocal microscopy to analyze vesicle-host cell interactions. Cultured A549 cells were incubated with FITC-labeled S470 vesicles for 6 hours at 37°C, and plasma membranes were stained with AF633-wheat germ agglutinin (WGA) to visualize cell boundaries. At 37°C, vesicle fluorescence appeared to be mostly internal and concentrated in a perinuclear region of the cell (Fig. 2B). Very little vesicle association was observed for incubations maintained at 4°C (Fig. 2C). Thus, both binding and internal localization of S470 vesicles was affected at the lower temperature.

**Figure 3:**
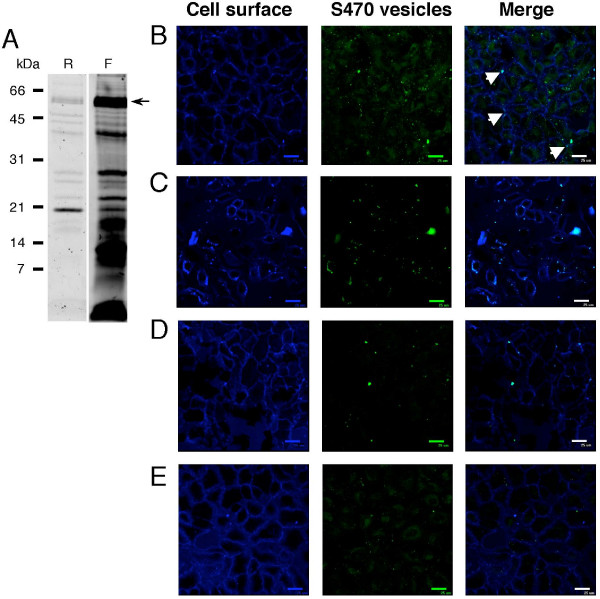
**Vesicle components are internalized by lung cells, and internalization is inhibited by hypertonic sucrose and cyclodextrins**. A, SDS-PAGE gel profiles of S470 vesicles before and after AF488 labeling. Total protein in unlabeled vesicles was visualized after SYPRO Ruby staining of the gel (R). AF488-labeled proteins were visualized by placing the unstained gel on a UV lightbox (F). The migration of molecular weight standards (kDa) and PaAP (arrow) is indicated. B, A549 cells incubated with 2.5 μg AF488-labeled S470 vesicles (green) for 1 h at 37°C. Cell surface was labeled using biotin and AF633-streptavidin (blue), fixed in 2% paraformaldehyde, and visualized by confocal microscopy. A549 cells were pretreated with 10 mM methyl-β-cyclodextrin (C), 10 mM α-cyclodextrin (D), or 0.45 M sucrose (E), for 30 minutes, and then incubated with 2.5 μg AF488-labeled S470 vesicles (green) for 1 h at 37°C. Cell surface was labeled using biotin and AF633-streptavidin (blue), fixed in 2% paraformaldehyde, and visualized by confocal microscopy. Bars indicate 25 μm.

To further confirm vesicle internalization, vesicles were labeled using AF488 instead of FITC to maximize fluorescence and minimize the effects of photobleaching. Many vesicle components are labeled by this reagent (Fig 3A), indicating that the fluorescent signal would not track merely a single component. After incubating AF488-S470 vesicles with A549 cells for 1 h at 37°C, the surface of cell monolayers was labeled with a membrane-impermeable biotin. The biotinylated surface was then detected using AF633-streptavidin and cell fluorescence was visualized by confocal microscopy. As a result, surface-exposed vesicles appear white and internalized vesicles appear green in an overlay of streptavidin and vesicle fluorescence. After a 1 hour incubation with A549 cells, mainly green, perinuclear fluorescence was observed (Fig 3B), with only a few white, surface localized vesicles (indicated by arrows, Fig 3B), indicating that S470 vesicles are internalized by lung cells.

To investigate the mode of *P. aeruginosa *vesicle internalization, we treated cells with common inhibitors of endocytic pathways. Filipin, chlorpromazine, cytochalasin D, and NiCl_2 _did not inhibit uptake (data not shown). Pre-treatment of cells with methyl-β-cyclodextrin (MβCD), which removes cholesterol from membranes, inhibited vesicle uptake, however, preincubation with methyl-α-cyclodextrin, which typically is used as a negative control for MβCD, inhibited vesicle uptake as well (Fig. 3C and 3D). Inhibition of vesicle uptake was also achieved using hypertonic sucrose (Fig 3E). In parallel control incubations, we pretreated vesicles with hypertonic sucrose or cyclodextrins instead of pretreating the lung cells. In these controls, vesicles were still readily internalized (data not shown), indicating that the inhibition of vesicle uptake was due to effects on the lung cells and not on the vesicles themselves.

**Figure 4:**
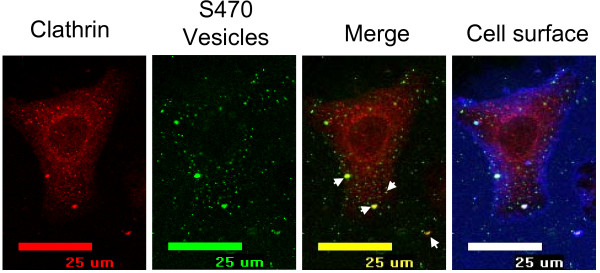
**Vesicles rarely co-localize with surface-associated clathrin**. AF488-S470 vesicles (2.5 μg) were incubated with A549 cells for 1 h at 37°C. Cell surface was labeled using biotin and AF633-streptavidin (blue). Cells were washed, fixed, permeabilized, and probed with mouse anti-clathrin antibodies and AF555-labeled goat anti-mouse secondary antibody. Arrows indicate very occasional colocalization of clathrin and vesicle fluorescence at the cell surface.

Since we observed the greatest effect on vesicle internalization using hypertonic sucrose and MβCD, which impair clathrin-coated pit formation and invagination, respectively [28,29], we next investigated whether vesicles would colocalize with clathrin. We observed a few locations where vesicle fluorescence colocalized with clathrin on the cell surface, however vesicle fluorescence did not co-localize with any intracellular clathrin (Fig 4). Vesicles did not colocalize with any caveolin, however it should be noted that very little caveolin was visualized in the A549 cells, consistent with reports of low levels of caveolin-1 expression in these cells [30,31] (data not shown). These data suggest that vesicles may be associated with clathrin-coated pits, but only transiently, at an early stage in the active uptake process.

### Internalized vesicle components colocalize with the endoplasmic reticulum

**Figure 5:**
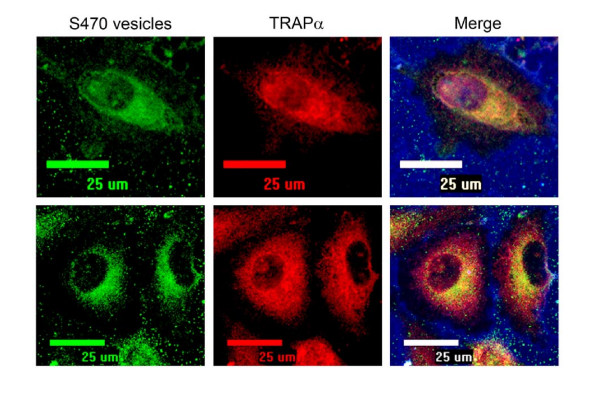
**Vesicles co-localize with the endoplasmic reticulum marker TRAPα**. AF488-S470 vesicles (2.5 μg) were incubated with A549 cells for 1 hour at 37°C. Cell surface was labeled using biotin and AF633-streptavidin (blue). Cells were washed, fixed, permeabilized with 0.015% digitonin to release cytoplasm, and probed with anti-TRAPα primary antibody and AF555-labeled secondary antibody.

We repeatedly observed internalized vesicle-associated fluorescence localized to a perinuclear region. We examined whether vesicles were trafficked to the same compartments as transferrin and cholera toxoid (CTB). Only transferrin and CTB that were perinuclear colocalized with internalized vesicles, whereas the majority of cytosolic compartments containing transferrin and CTB did not [see Additional file 1]. To determine whether this perinuclear region corresponded to the endoplasmic reticulum (ER), we treated cells with the glycoside digitonin, which, at low concentrations, permeabilizes the plasma membrane and releases cytosolic proteins but preserves the ER membrane [32,33]. After digitonin treatment, cells that had lost the cytoplasmic marker, β-tubulin, still retained a perinuclear halo of vesicle-associated fluorescence (data not shown). In these treated cells, vesicle fluorescence clearly colocalized with the integral ER membrane protein TRAPα (Fig. 5). These data suggest that internalized vesicle components traffic to the ER within 1 hour of exposure.

### PaAP promotes vesicle association with human respiratory epithelial cells

**Figure 6:**
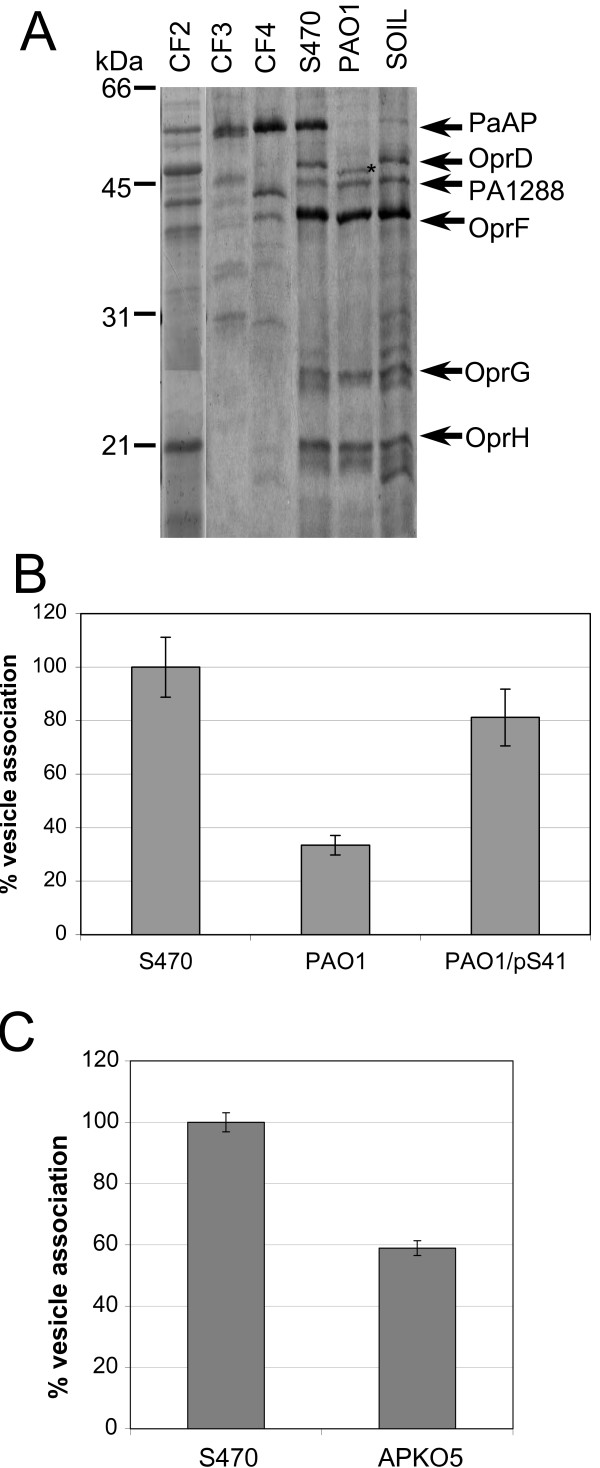
**PaAP is abundant and active in vesicles from CF strains and promotes the association of P. aeruginosa vesicles with lung cells**. A, Purified vesicles (approximately 10 μg) were TCA-precipitated and analyzed using SDS-PAGE and Coomassie staining. Previously identified proteins in PA01 vesicles and CF2 vesicles are indicated, and (*) highlights the lower molecular weight form of OprD found in PA01 [8]. The migration of molecular weight standards is indicated (kDa). B and C, Purified vesicles from the indicated strains (2.5 μg protein/well) were incubated (24 h, 37°C) with confluent monolayers of A549 cells (5 × 10^4^/well) and vesicle-host cell association was compared with S470 vesicle association within each experimental set. SEM is indicated; n = 2 in triplicate.

We wondered whether host cell association depended on PaAP, one of the major protein components of vesicles derived from CF isolates (Fig 6A). Quantitative 2D-DIGE revealed PaAP is at least 65-fold enriched in S470 vesicles compared with PAO1 vesicles [8]. To test whether PaAP on vesicles contributed to host cell association, we overexpressed PaAP in PAO1 (PAO1/pS41) and constructed a deletion in the aminopeptidase in strain S470 (S470APKO5). We confirmed that purified PAO1/pS41 vesicles were enriched in PaAP compared with PA01 vesicles and that S470APKO5 did not contain detectable amounts of PaAP [see Additional file 2]. Purified PAO1/pS41 vesicles associated with A549 cells more than twice as much as PA01 vesicles, whereas S470APKO5 vesicles associated 40% less with the lung cells than S470 vesicles (Fig. 6B, C). Unfortunately, complementation of S470APKO5 was not successful since vesicles from S470APKO5 expressing PaAP from pS41 contained approximately 10-fold less PaAP and had 10-fold less aminopeptidase activity than S470 vesicles [see Additional file 3, parts A and B]. Induction of PaAP expression in S470APKO5 did not help correct the complementation defect and increase the level of vesicle-bound PaAP, although the total amount of PaAP in the supernatant was equivalent to that of S470 [see Additional file 3, part C]. As a result, it was not surprising that S470APKO5/pS41 vesicles associated with host cells to approximately the same extent as those from APKO5 (data not shown). Collectively, these data support a dose-dependent contribution of PaAP to the association of vesicles with host cells.

## Discussion

With these results, we have revealed several facets of interactions between *P. aeruginosa *vesicles and human lung epithelial cells. We have demonstrated that *P. aeruginosa *vesicles are internalized by epithelial cells and trafficked intracellularly so that vesicle components accumulate in the ER. We have also shown that PaAP, an enzyme more abundant in vesicles produced by many CF isolates compared with non-clinical isolates, significantly contributes to the interaction of *P. aeruginosa *vesicles with host cells.

Internalization by host cells has been reported to occur for outer membrane vesicles from numerous species. For instance, our lab has shown previously that ETEC vesicles are internalized in an LT-dependent fashion via ganglioside G_M1 _in caveolin-enriched lipid rafts of epithelial cells [20]. Additionally, fluorescent beads coated with *Porphyromonas gingivalis *vesicles also colocalized with caveolin and G_M1 _and were internalized by HeLa cells in a cholesterol and dynamin-dependent manner [34]. Our observations regarding the temperature-dependent extent and location of vesicle-associated fluorescence in host cells and decreased fluorescence in host cells upon pretreatment with methyl-β-cyclodextrin (which disrupts caveolae, lipid rafts, as well as clathrin-coated pit-mediated entry pathways) suggested that S470 vesicles were also internalized.

In contrast to other examples of internalized vesicles, *P aeruginosa *vesicles appear to enter host cells via multiple pathways. Hypertonic media, which impairs clathrin coated pit formation, did significantly decrease vesicle internalization and some surface-bound vesicles were found colocalized with clathrin. However, neither treatment with filipin, which disrupts lipid rafts, nor chlorpromazine, which blocks clathrin-coated pits, decreased vesicle internalization significantly. It should also be considered that *P. aeruginosa *vesicles could fuse with the epithelial cells and that vesicle membrane components are subsequently internalized by plasma membrane trafficking while lumenal components are liberated into the host cell cytosol. Evidence of fusion of vesicles with the plasma membrane has been presented for *Actinobacillus actinomycetemcomitans *vesicles [13]: Confocal microscopy of HL60 cells coincubated with these vesicles showed immediate and strong labelling, primarily at the plasma membrane. We did not observe strong perimeter labelling of host cells with *P. aeruginosa *vesicles (Fig 2B). In fact, when we blocked active transport with hypertonic sucrose, we found a significant decrease in vesicle-associated fluorescence, not accumulation of fluorescence at the cell periphery (Fig 3E). Thus, our data support a model where *P. aeruginosa *vesicles do not fuse to the plasma membrane, but instead bind and are internalized.

We observed an increase in human lung epithelial cell-associated fluorescence over time. This result is consistent with either vesicle attachment causing receptor upregulation, or continuous vesicle binding, internalization, recycling of vesicle receptors to the cell surface. These characteristics are similar to the behavior of enterotoxigenic *E. coli *vesicles with intestinal epithelial cells [14]. Further experiments using different inhibitors, markers, and cell lines, will be necessary to definitively identify the host cell factors critical to *P. aeruginosa *vesicle entry. In relation to CF-related research, it would be particularly interesting to see whether the interactions depend on functional and properly localized CFTR. Ceramide-rich rafts containing clusters of the CFTR and CD95 have been implicated as the means for internalization of whole *P. aeruginosa*. These rafts are disrupted by MβCD, and thus in light of our MβCD treatment results, they present a potential route for vesicle internalization [35].

Once internalized, vesicles may be sequestered, degraded, or their components may be dispersed, with their membranes disrupted and/or fused with the endocytic membrane. In the case of *S. flexneri *vesicles, for instance, vesicle lumenal content was found in the host cell cytosol after vesicles were phagocytosed to a non-acidified compartment by Henle 407 epithelial cells [36]. We show that *P. aeruginosa *vesicle-associated intracellular fluorescence is concentrated to bright puncta and do not encounter an acidified compartment, since vesicle-associated FITC fluorescence (which is pH sensitive) is not quenched, even in long incubations (Fig 1). Notably, a significant amount of vesicle-associated fluorescence colocalized with the integral ER membrane protein TRAPα, even after a relatively brief incubation time. Transferrin and CT eventually route to the ER, and indeed, those pools of Transferrin and CT that had reached the ER colocalized with the vesicle fluorescence. None of the currently identified *P. aeruginosa *vesicle proteins have an ER retention sequence to direct the trafficking of these bacterial factors to the ER (such as the case for LT which has RDEL at its C-terminus). Since intracellular trafficking of S470APKO5 vesicles was not noticeably different from S470 vesicles (data not shown), internalized vesicle trafficking appears to be PaAP-independent. In all, many questions remain regarding the trafficking of *P. aeruginosa *vesicle membrane and lumenal content after endocytosis, and this area deserves further exploration.

In some cases the factor on bacterial vesicles responsible for host cell binding has been identified as a virulence factor [9]. For example, the heat-labile enterotoxin (LT) is bound to the surface of ETEC vesicles, and vesicle-bound LT mediates vesicle binding to cultured eukaryotic cells via the LT receptor, ganglioside G_M1 _[11,14]. In contrast, leukotoxin transported in *A. actinomycetemcomitans *vesicles was not responsible for vesicle association with HL60 cells [13]. We have found that PaAP also is located on the vesicle surface (preliminary data), and that host cell association correlated with PaAP levels on the vesicles. Strains overexpressing PaAP or deleted in PaAP, respectively, produced vesicles that associated to a greater or lesser extent than vesicles from the corresponding isogenic parent strains. A direct correlation between vesicle association and PaAP levels also held for strains naturally expressing PaAP at different levels. PaAP expression is highly regulated and typically does not occur until stationary phase [37-40]. This was true for our cultures of PAO1, and as a result PaAP was nearly absent from PAO1 vesicles purified from late log-phase cultures (see Fig 6 and [Additional file 2, Part A]). In contrast, strain S470 begins to express PaAP in late log phase, therefore PaAP was enriched in the late log-phase S470 vesicles (see Fig 6 and [Additional file 2, Part A]). Correspondingly, PAO1 vesicles associated 3–4 fold less than S470 vesicles (Fig 1). Thus, the degree of bacterial vesicle-host cell interaction can depend on the growth phase of the parent bacterium. Despite the importance of PaAP, it is not the only factor governing host cell association since association by S470APKO5 vesicles was only reduced by 40% compared with S470 vesicles. The conclusion that *P. aeruginosa *vesicles can utilize numerous internalization pathways is consistent with our finding that factors other than PaAP are involved in vesicle-host cell association.

We describe that PaAP expression in *trans *failed to complement the PaAP deletion with regards to the ability to obtain WT levels of vesicle-localized PaAP, and hence its ability to restore WT levels of vesicle association with host cells. Complemented PaAP was expressed and secreted into the culture supernatant at WT levels, however it was not found in the vesicle-associated fraction [see Additional file 3]. In fact, overexpression of PaAP in the null mutant resulted in reduced viability (unpublished data). This lack of functional complementation is not unprecedented. Two other secreted *P. aeruginosa *proteases (LasA and protease IV) have knockout phenotypes which could not be complemented by expression of the gene from a plasmid or even from a chromosomal insertion [41-43]. The lack of complementation by the plasmid-expressed PaAP in the APKO5 PaAP knockout strain demonstrates the likelihood of a fine-tuned regulatory process that is critical for optimal PaAP expression, processing, stability, and/or secretion. Indeed, PaAP has been found to undergo complex post-translational processing ((D. FitzGerald, personal communication, and [44]).

Since vesicle-associated PaAP has activity as a zinc-dependent protease, PaAP could act as a proteolytic factor that exposes vesicle receptors on the host cell surface. In an attempt to test this, we constructed a mutant PaAP which lacked active site residues however it was not secreted (preliminary data). Interestingly, this suggests PaAP must bind zinc for it to fold correctly and folding is critical for export of Type 2 secretory pathway substrates. As a result, we have not yet been able to test whether PaAP activity is important in mediating host cell interactions, internalization, or trafficking.

**Figure 7:**
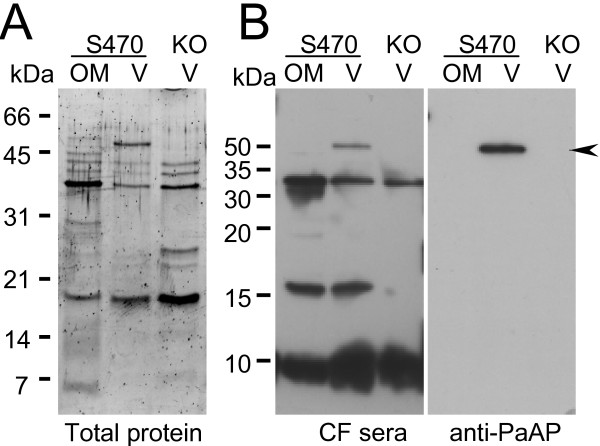
**CF patients produce antibodies to PaAP**. Purified outer membranes (OM) from S470 and vesicles (V) from S470 and S470APKO5 (KO) (2 μg) were separated by SDS-PAGE and stained with SYPRO Ruby (A) or transferred to PVDF and immunoblotted using sera from a CF patient and then reblotted with anti-PaAP (*B*). Molecular weight standards (kDa) and the migration of PaAP (arrow) are indicated.

We discovered several characteristics of PaAP expression relevant to the virulence of *P. aeruginosa *in the CF lung. First, strains taken from patients with CF express PaAP abundantly. Second, we found that more PaAP is detectable in vesicles produced by PA01 that contain the β-lactamase-resistant vector pMMB66EH than in those produced by PA01 [see Additional file 2, part A]. The association of these vesicles with lung cells was consistent with the previously described trend: PAO1/pMMB66EH vesicles associated with host cells to a greater extent compared to PA01 vesicles [see Additional file 2, part B]. The increase in PaAP expression in this case appears to be due to the presence of antibiotics in the media, which is particularly relevant considering that bacteria in the CF lungs are frequently exposed to antibiotics. Finally, sera from CF patients contained antibodies to several vesicle proteins, and a subset of patients (3 out of 13) produced antibodies to PaAP indicating that PaAP is expressed and secreted in CF patients (Fig. 7). These findings suggest that the conditions present in infected CF lungs promote upregulation of *P. aeruginosa *PaAP and production of vesicles that contain PaAP.

## Conclusion

Purified *P. aeruginosa *vesicles associate with human lung cells and are internalized in a time- and dose-dependent manner. Vesicles from a CF isolate exhibit greater association with lung cells than vesicles from a lab strain. Vesicle internalization is temperature-dependent and inhibited by hypertonic sucrose and cyclodextrins. Vesicles also appear to be very transiently associated with clathrin-coated pits as part of an active uptake process. After internalization, vesicle components were found to colocalize with the ER. Tested CF isolates of *P. aeruginosa *abundantly secrete PaAP, an aminopeptidase which is a major contributor to lung cell association. Therefore, our results suggest that *P. aeruginosa *vesicles can interact with and be internalized by lung epithelial cells and thereby contribute to the inflammatory response during infection.

## Methods

### Bacterial strains and reagents

*P. aeruginosa *strains used were the laboratory strain PA01 (Pf1 phage-cured from our lab collection), the soil isolate ATCC 14886 (American Type Culture Collection, isolated prior to 1958), and minimally passaged, non-mucoid cystic fibrosis clinical isolates CF2, CF3, CF4, and S470 (Duke University Hospital). A549 human lung epithelia carcinoma cells were grown according to ATCC specifications in Kaighn's F-12K media containing 10% fetal bovine serum plus penicillin/streptomycin/fungizone. Human bronchial epithelial (HBE) cells were derived from anonymous healthy human volunteers. HBE cells were maintained in Bronchial Epithelial Cell Growth Media supplemented with thyroid extract. Unless indicated, all reagents were purchased from VWR.

### Construction of PA01 overexpressing PaAP (PA01/pS41)

The PA2939 gene encoding PaAP was amplified from strain S470 using the primers given in Table 1, which added an EcoRI site to the 5' end of the sequence a HindIII site to the 3' end of the sequence. The gene was subcloned into pBluescript and then moved to pMMB66EH (provided by Erich Lanka) to make plasmid pS41. Plasmid pS41 was moved into PA01 by triparental mating as described [45], using HB101/pS41 as the donor strain and MK616 (containing pRK2013) as the helper strain. Successful conjugants were selected on Pseudomonas Isolation Agar (PIA) supplemented with carbenicillin (200 μg/ml) and verified by restriction digestion of miniprepped DNA.

**Table 1:** Primers used in these study (*5' to 3' sequence*)

	**PA2939**
	
expression forward	TTACCGGAATTCATGAGCAACAAGAACA
expression reverse	AACGGCAAGCTTTTACTTGATGAAGTCG
KO up forward	TGTAACTAGTATGGTCAGCACATGTTGCA
KO up reverse	GCCAGGGATGCGGCGGAATTCGAGAGGGCGAGGGCG
KO down forward	CGCCCTCGCCCTCTCGAATTCCGCCGCATCCCTGGC
KO down reverse	CTGACCTCGAGTTACTTGATGAAGTCGTGAC
	
	**Tet**^**R **^**cassette from pACYC184**
	
EcoRI Tet forward	GGTTATGAATTCGGTAGCTCAGAGAACCCTTCG
EcoRI Tet reverse	GTGTTAGAATTCGATATGTTCTGCCAAGGGTT
Xho Tet forward	CCGGCTCGAGGGTAGCTCAGAGAACCTTCG
Xho Tet reverse	CCGGCTCGAGGATATGTTCTGCCAAGGGTT

### Construction of PA2939 knockout in S470 (strain APKO5)

The PA2939 knockout vector (pAPKO) was constructed by interrupting the PA2939 sequence with a Tet cassette. DNA sequence starting from approximately 500 bp upstream of the PA2939 start codon to 30 bp into PA2939 was amplified by PCR using the "up" primers given in Table 1, which added a SpeI site to the 5' end of the DNA and mutated the 3' end to contain an EcoRI site. The remainder of the PA2939 sequence was amplified with the "down" primers given in Table 1, which mutated the 5' end to contain an EcoRI site and added an XhoI site to 3' end. The Tet cassette was amplified from plasmid pACYC184 using primers given in Table 1 that added EcoRI sites to both ends. The three pieces were combined sequentially using the pDrive subcloning vector (Qiagen). The final construct was cut out of pDrive using SpeI and XhoI sites and inserted into the MCS of pJQ200SK (Gm^R^, SacB) to make plasmid pAPKO.

Triparental mating was used to introduce pAPKO into strain S470 using HB101/pAPKO as the donor strain, and MT616 as the helper strain. Successful conjugants were first selected on 1/2 PIA Tet (200 μg/ml) and Gm (20 μg/ml). Bacterial colonies that had undergone homologous recombination with the DNA containing the interruption of PA2939 were then counter-selected for resistance to Tet and sensitivity 5% sucrose and Gm. Knockout S470APKO5 was verified by PCR amplification of the interrupted PA2939 sequence, sequencing of the interrupted gene, and immunoblotting with anti-PaAP.

S470APKO5 was complemented with vector pS41 or empty vector pMMB66EH by triparental mating, as described above. Complementation was verified by PCR, restriction digests of plasmid DNA, and aminopeptidase detection by immunoblot and activity.

### Vesicle isolation and purification

Vesicles were purified from a method adapted from Horstman and Kuehn [11]. Bacteria were grown in LB broth overnight to early stationary phase. Cells were removed by pelleting (10,000 × *g*, 10 min). Supernatants were concentrated via a 100-kDa tangential filtration concentration unit (Pall-Gellman) to approximately 1/25^th ^their original volume. The retentate was collected and centrifuged (6000 × *g*, 10 min) and then filtered through a 0.45 μm Durapore PVDF filter (Millipore) to remove remaining bacterial cells. Vesicles were obtained from the cell-free supernatant by one of two methods. In the first method, the vesicles were pelleted (39,000 × *g*, 1 h), resuspended in 50 mM HEPES, pH 6.8 (HEPES), and adjusted to 45% Optiprep (Greiner) in 10 mM HEPES/0.85% NaCl, pH 7.4 (HEPES-NaCl) (weight/weight). In the second method, the vesicles were precipitated with 71–75% ammonium sulfate (4°C, for at least 3 h), pelleted (10,000 × *g*, 20 min), dialyzed overnight with HEPES, concentrated (50 kDa MWCO Centriplus, Millipore), and adjusted to 45% Optiprep/HEPES-NaCl. Optiprep gradients were layered over the 2 ml crude vesicle samples as follows: For PAO1: 2 ml 40%, 2 ml 35%, 3 ml 30%, 2 ml 25%, 1 ml 20%; for Soil: 2 ml 40%, 2 ml 35%, 2 ml 30%, 2 ml 25%, 2 ml 20%; for CF isolates: 2 ml 40%, 2 ml 35%, 4 ml 30%, 2 ml 20% Optiprep/HEPES-NaCl by weight. Gradients were centrifuged (100,000 × *g*, 16 h) and 1 ml fractions removed from the top. A portion of each fraction was visualized by 15% SDS-PAGE. Pure vesicles were recovered from pooled peak fractions by dialyzing overnight against HEPES and pelleting (150,000 × *g*, 1 h). Vesicles were checked for sterility by culturing 5–50 μL on LB agar overnight at 37°C. Contaminated vesicles were filtered through 0.45 μm Microcon spin filters (Millipore) and recultured on LB plates.

### Fluorescent labeling of vesicles

Purified vesicles were fluorescently labeled by incubating with fluorescein isothiocyanate (FITC) reagent (Sigma)(1 μg FITC/μg vesicle protein in 100 mM NaCl/50 mM Na_2_CO_3_, pH 9.2), for 2 h at 25°C with mixing, or with AlexaFluor-488 succinimidyl ester (AF488, Invitrogen, in 0.1 M Na_2_CO_3_, pH 9) according to manufacturer's instructions, for 1 h at 25°C with mixing. Free FITC and AF488 were removed from labeled vesicles by washing three times in HEPES (150,000 × *g*, 30 min). Labeled vesicles were checked for sterility and filtered through 0.45 μm PVDF spin filters when necessary.

### Vesicle association assays

FITC-labeled vesicles (2.5 μg per well) were incubated with confluent monolayers (approx. 5 × 10^4 ^cells per well) of A549 human lung epithelia or HBE cells in serum-free media in 96-well plates (Costar) for 15 min to 24 h at 37°C or 4°C. All incubation conditions were done in triplicate. Cells were washed twice with PBS and then solubilized in 100 μ1 1% Triton X-100 in PBS. Fluorescence was quantitated using a FLUOstar Galaxy or FLUOstar Optima fluorometer (BMG Labtechnologies). A standard curve to correlate fluorescence measured in test wells to ng of vesicles was generated by adding purified FITC-labeled vesicles (0.5 ng–250 ng) from each strain to cells and immediately solubilizing the cells. Statistics were calculated using single-factor ANOVA.

### Confocal microscopy

All fluorescence microscopy reagents were purchased from Molecular Probes/Invitrogen unless otherwise stated. A549 human lung epithelia (1 × 10^5 ^cells per well) were seeded in LabTekII 8-well glass chamber slides (VWR) and incubated with fluorescently labeled vesicles (2.5 μg per well) in serum-free media for 1–6 h at 37°C or 4°C. When indicated, AlexaFluor-555 transferrin (25 μg/ml) or AlexaFluor-555 cholera toxin B subunit (10 μg/ml) were added to cells five minutes prior to the addition of vesicles. For inhibition experiments, cells were pretreated with inhibitors (methyl-β-cyclodextrin, 10 mM; methyl-α-cyclodextrin, 10 mM; sucrose, 0.45 M; chlorpromazine, 1 μg/ml; filipin, 5 μg/ml; cytochalasin D, 1 μg/ml; NiCl_2_, 2 mM) for 30 min, and the inhibitors remained in the media during incubation with vesicles. All subsequent steps were carried out on ice and ice-cold Dulbecco's phosphate-buffered saline (PBS) was used for washes.

Following incubation with vesicles, cells were washed twice to remove unbound vesicles. Cell exteriors were labeled in one of two ways, as indicated in figure legends: 1) Cells were incubated with AF633-conjugated wheat germ agglutinin (WGA; 25 min, on ice) and washed twice, or 2) Cells were incubated with 6-((biotinoyl)amino)hexanoic acid, succinimidyl ester (Biotin-X, SE; 10 min, on ice), washed twice, and then incubated with AF633-conjugated streptavidin (15 min, on ice) and washed twice. Cells were then fixed in 2% paraformaldehyde, mounted with ProLong AntiFade reagent, and visualized on a Nikon Eclipse TE200.

### Immunofluorescence

Clathrin and caveolin immunofluorescence was performed essentially as described [14]. Following incubation with vesicles, monolayers were washed, cell exteriors were labeled with Biotin-X, SE/AF633-Streptavidin and fixed as described above. Fixed cells were washed, permeabilized (0.1% Triton X-100 in Hanks buffer; 15 min, 25°C), blocked (5% goat serum and 0.1% bovine serum albumin in permeabilization buffer; 20 min, 25°C), incubated with mouse anti-caveolin-1 or anti-clathrin antibodies (BD Biosciences; 2.5 μg/ml in permeabilization buffer; 1 h, 25°C), washed, and then labeled with AF555-conjugated goat anti-mouse secondary antibody (μg/ml in permeabilization buffer; 30 min, 25°C), and washed. Following incubation with secondary antibodies, slides were mounted and visualized as described above.

For TRAPα and tubulin immunofluorescence, fixed monolayers were permeabilized in PBS supplemented with 1 mM DTT, 1 mM PMSF, and 0.015% digitonin (to release cytoplasmic contents) for 5 min. Permeabilized cells were blocked with 1% BSA in PBS (30 min, on ice), incubated with rabbit anti-TRAPα or mouse anti-β-tubulin primary antibodies (2 μg/ml, in blocking buffer, 1 h, on ice), washed, and incubated with AF555-conjugated goat anti-mouse or anti-rabbit secondary antibodies (30 min, on ice). Following incubation with secondary antibody, slides were mounted and visualized as described above.

### Leucine aminopeptidase assay

Assays were performed using the substrate Leu-*p*-nitroanilide (0.6 mM in 50 mM Tris-HCl, 1 mM CaCl_2_, pH 8.3) as described previously [44]. Samples were preincubated with 0.5 mM dithiothreitol (30 min, 25°C) in controls experiments (data not shown) to demonstrate specificity. The production of p-nitroaniline (pNA) was monitored at 405 nm.

### Detection of PaAP antibodies in sera from CF patients

Outer membranes were purified as described [46,47]. Briefly, cells were harvested in stationary phase, resuspended (20% sucrose in 30 mM Tris, pH 8), treated with DNase I and RNaseA and broken by French Press. Membranes were separated using a sucrose gradient. Purified S470 outer membrane and vesicles (2 μg) were separated by SDS-PAGE and SYPRO Ruby protein stained or transferred to PVDF, immunoblotted using sera (1:10 dilution) from anonymous CF patients or anti-PaAP antibodies, and developed with SuperSignal (Pierce).

## Authors' contributions

S.J.B. was responsible for designing and carrying out the experiments, M.J.K. was responsible for overseeing the research design and funding, both authors participated in data interpretation and writing of the manuscript.

## Supplementary Material

Additional file 1 **Vesicles primarily colocalize with CT and transferrin in peri-nuclear regions.** The data show fluorescently labeled S470 vesicles colocalize with CT and transferrin in perinuclear regions of A549 cells. Click here for file

Additional file 2 **PaAP contributes to the cell association of vesicles in a dose-dependent manner.** The data show the amount of PaAP on vesicles correlates with the amount of vesicle association with A549 cells. Click here for file

Additional file 3 **Vesicle expression and activity of S470APKO5 complemented with plasmid-expressed PaAP.** The data show the lack of PaAP activity in the APKO5 strain, the correlation between secreted aminopeptidase activity of the complemented strain with the amount of PaAP secreted, and that induced, plasmid-expressed PaAP in APKO5 is secreted to the same extent as S470 but is not vesicle-associated. Click here for file
