# Supplementary material for: Pseudomonas aeruginosa vesicles associate with and are internalized by human lung epithelial cells
Source: BMC Microbiol. 2009 Feb 3;9:26. doi: 10.1186/1471-2180-9-26 (PMC2653510; doi:10.1186/1471-2180-9-26)
Supplement: Additional file 1 — Vesicles primarily colocalize with CT and transferrin in peri-nuclear regions. The data show fluorescently labeled S470 vesicles colocalize with CT and transferrin in perinuclear regions of A549 cells. [file 1471-2180-9-26-S1.pdf]

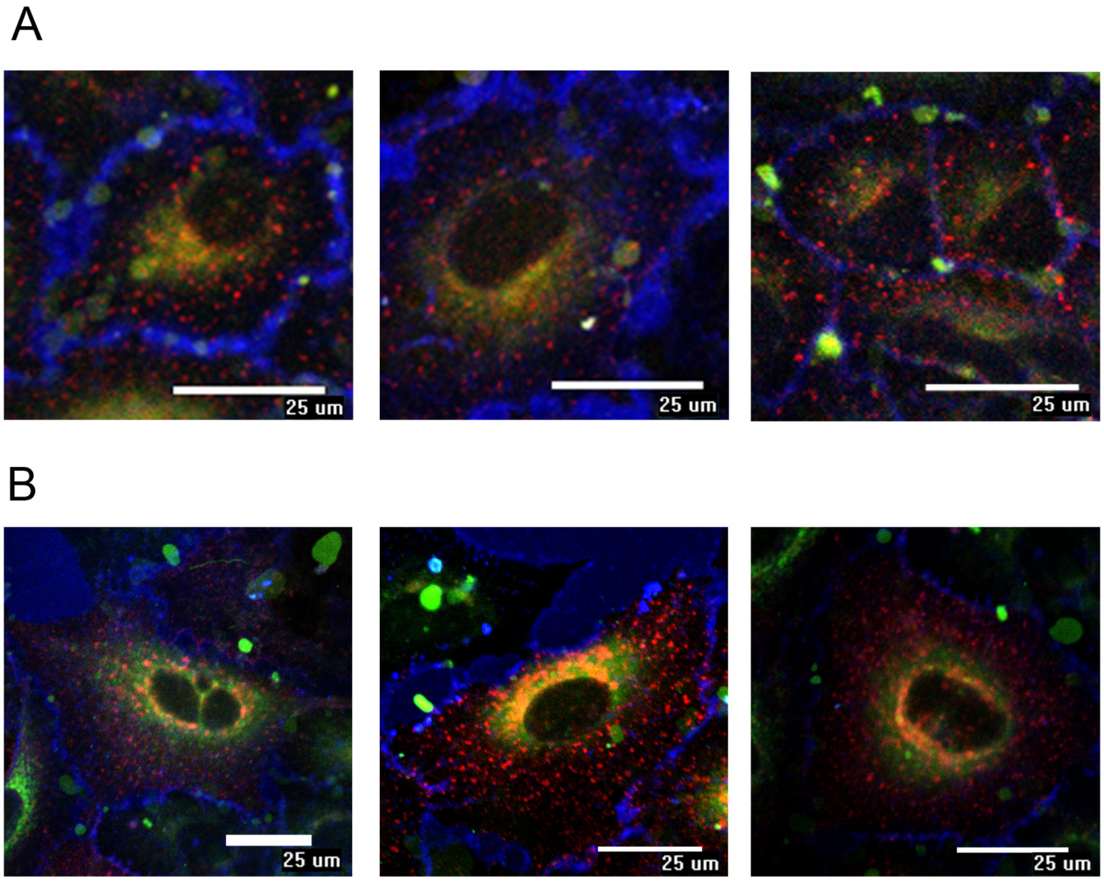

Additional File1. Vesicles primarily colocalize with CT and transferrin in peri-nuclear regions. A549 cells were incubated with 2.5 μg AF488-labeled S470 vesicles (green) and either 25 μg of AF555-transferrin (A) or 5 μg of AF555-CTB (B) (red) for 1 h at 37°C. Cell surface was labeled using biotin and AF633-streptavidin (blue), fixed in 2% paraformaldehyde, and visualized by confocal microscopy.
