# Supplementary material for: Pseudomonas aeruginosa vesicles associate with and are internalized by human lung epithelial cells
Source: BMC Microbiol. 2009 Feb 3;9:26. doi: 10.1186/1471-2180-9-26 (PMC2653510; doi:10.1186/1471-2180-9-26)
Supplement: Additional file 2 — PaAP contributes to the cell association of vesicles in a dose-dependent manner. The data show the amount of PaAP on vesicles correlates with the amount of vesicle association with A549 cells. [file 1471-2180-9-26-S2.pdf]

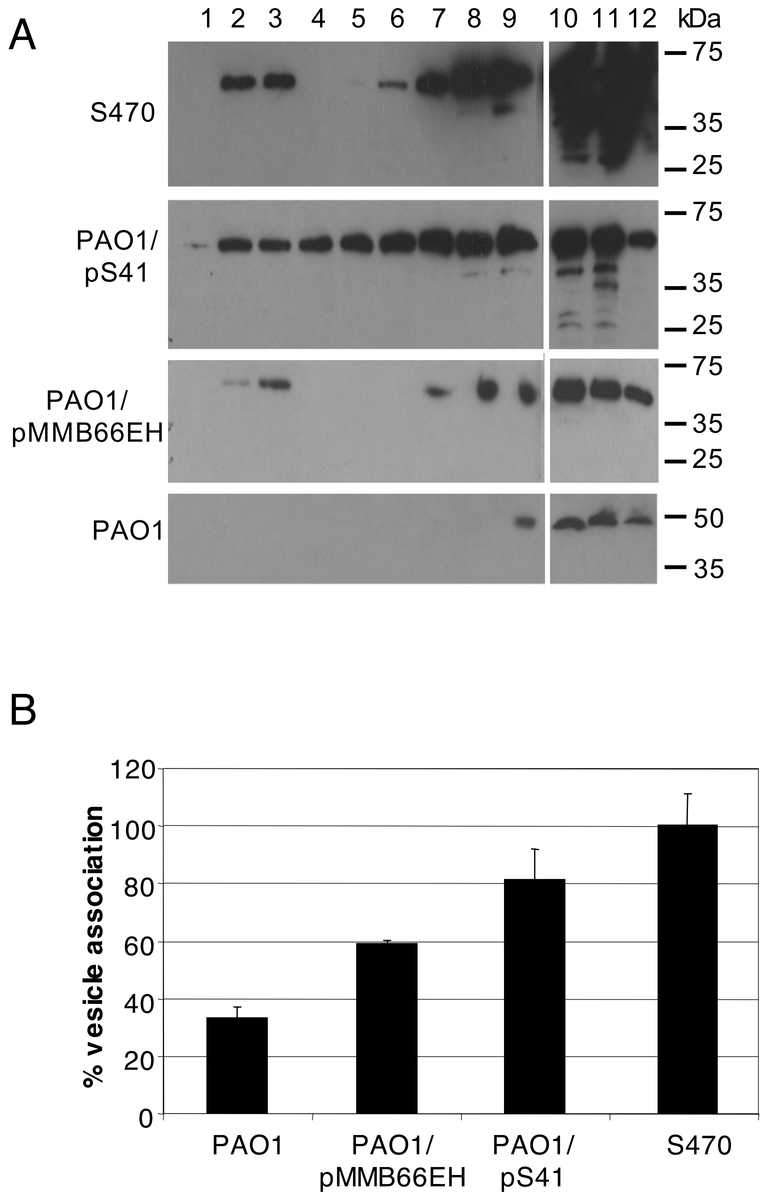

Additional File 2. PaAP contributes to the cell association of vesicles in a dose-dependent manner. A, Ammonium sulfate-precipitated supernatants from S470, PAO1 overexpressing PaAP (PAO1/pS41), PAO1 containing empty vector (PAO1/pMMB66EH), and PAO1 were fractionated on a density gradient. Fractions were immunoblotted for PaAP. Purified vesicles float to fractions 1-3. B, Purified vesicles from the indicated strains (2.5 μg protein/well) were incubated (24 h, 37°C) with confluent monolayers of A549 cells ($5x10^4$/well) and vesicle association was determined as described in Fig. 1 and compared with vesicle association of S470 vesicles within each experimental set. Assays were performed in triplicate; SEM is indicated for 2 separate experiments.
