# Supplementary material for: Pseudomonas aeruginosa vesicles associate with and are internalized by human lung epithelial cells
Source: BMC Microbiol. 2009 Feb 3;9:26. doi: 10.1186/1471-2180-9-26 (PMC2653510; doi:10.1186/1471-2180-9-26)
Supplement: Additional file 3 — Vesicle expression and activity of S470APKO5 complemented with plasmid-expressed PaAP. The data show the lack of PaAP activity in the APKO5 strain, the correlation between secreted aminopeptidase activity of the complemented strain with the amount of PaAP secreted, and that induced, plasmid-expressed PaAP in APKO5 is secreted to the same extent as S470 but is not vesicle-associated. [file 1471-2180-9-26-S3.pdf]

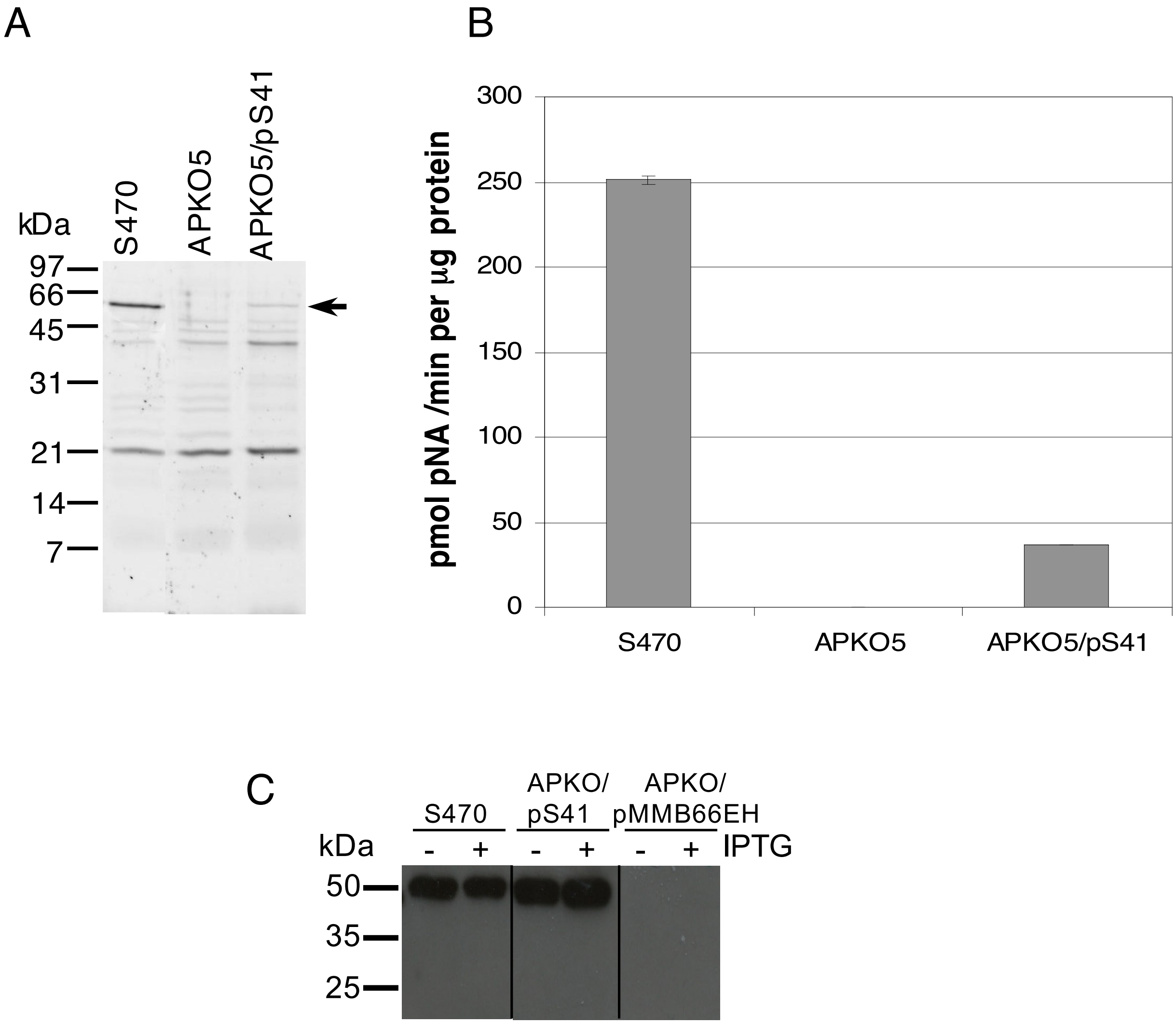

Additional File 3. Vesicle expression and activity of S470APKO5 complemented with plasmid-expressed PaAP. A, A, Purified vesicle fractions of S470, S470APKO5 (APKO5) and S470APKO5 overexpressing PaAP (APKO5/pS41) applied to SDS-PAGE and stained with SYPRO Ruby. **B.** Aminopeptidase activity of samples described in part A. Activity measurements were performed in duplicate, SEM is indicated for 2 separate experiments. C. PaAP secretion is not limiting in cell-free supernatants of complemented PaAP null mutant. Cultures of the indicated strains were diluted from uninduced overnight cultures and grown to an OD_{600} of 1.0-1.1 with or without 50 μM IPTG. At the given OD, 1ml of supernatant was 0.45 μm sterile-filtered and TCA-precipitated to 50 μl, 20 μl of which was loaded on the gel.
